# Perilipin-Mediated Lipid Droplet Formation in Adipocytes Promotes Sterol Regulatory Element-Binding Protein-1 Processing and Triacylglyceride Accumulation

**DOI:** 10.1371/journal.pone.0064605

**Published:** 2013-05-29

**Authors:** Yu Takahashi, Akihiro Shinoda, Norihiko Furuya, Eri Harada, Naoto Arimura, Ikuyo Ichi, Yoko Fujiwara, Jun Inoue, Ryuichiro Sato

**Affiliations:** 1 Department of Applied Biological Chemistry, Graduate School of Agricultural and Life Sciences, The University of Tokyo, Tokyo, Japan; 2 Department of Nutrition and Food Science, Ochanomizu University, Tokyo, Japan; Graduate School of Medicine, the University of Tokyo, Japan

## Abstract

Sterol regulatory element-binding protein-1 (SREBP-1) has been thought to be a critical factor that assists adipogenesis. During adipogenesis SREBP-1 stimulates lipogenic gene expression, and peroxisome proliferator-activated receptor γ (PPARγ) enhances perilipin (plin) gene expression, resulting in generating lipid droplets (LDs) to store triacylglycerol (TAG) in adipocytes. Plin coats adipocyte LDs and protects them from lipolysis. Here we show in white adipose tissue (WAT) of plin−/− mice that nuclear active SREBP-1 and its target gene expression, but not nuclear SREBP-2, significantly decreased on attenuated LD formation. When plin−/− mouse embryonic fibroblasts (MEFs) differentiated into adipocytes, attenuated LDs were formed and nuclear SREBP-1 decreased, but enforced plin expression restored them to their original state. Since LDs are largely derived from the endoplasmic reticulum (ER), alterations in the ER cholesterol content were investigated during adipogenesis of 3T3-L1 cells. The ER cholesterol greatly reduced in differentiated adipocytes. The ER cholesterol level in plin−/− WAT was significantly higher than that of wild-type mice, suggesting that increased LD formation caused a change in ER environment along with a decrease in cholesterol. When GFP-SREBP-1 fusion proteins were exogenously expressed in 3T3-L1 cells, a mutant protein lacking the S1P cleavage site was poorly processed during adipogenesis, providing evidence of the increased canonical pathway for SREBP processing in which SREBP-1 is activated by two cleavage enzymes in the Golgi. Therefore, LD biogenesis may create the ER microenvironment favorable for SREBP-1 activation. We describe the novel interplay between LD formation and SREBP-1 activation through a positive feedback loop.

## Introduction

In mature adipocytes, TAGs are stored as an energy source within LDs surrounded by a phospholipid monolayer and plin, which not only protects LDs but also regulates lipolysis by controlling lipase access to them in a hormone-regulated manner. Plin−/− mice with WAT containing smaller LDs surrounded by adipose differentiation-related protein (ADRP), a plin family member, exhibit a lean phenotype and are resistant to diet-induced obesity [1]. TAG is believed to be synthesized and released between the leaflets of the bilayer membrane of the ER. Once TAG accumulates in the membrane above a threshold level, LDs are released into the cytoplasm by budding. The finding that several proteins, mainly localized in ER, decorate LD surfaces supports a tight connection between ER and LDs. However, little is known about the precise molecular mechanism of LD biogenesis in adipocytes [2].

SREBP-1 was discovered as a transcription factor regulating low density lipoprotein receptor gene expression [3], [4] and coincidentally as adipocyte determination- and differentiation-dependent factor 1 [5]. It was later reported to be involved in regulation of lipogenic rather than cholesterol metabolism gene expression. SREBP-1 and -2 form a complex with the SREBP cleavage-activating protein (SCAP) binding to COPII proteins, travelling from ER to the Golgi complex [6]. SREBPs are then processed by 2 proteases, S1P and S2P, liberating the active N-terminal domain, which enters the nucleus and activates their target genes. When excess cholesterol accumulates in the ER membrane, the SREBP/SCAP complex binds to the ER membrane protein Insig and remains in ER. Although both SREBPs are activated through the same processing pathway, the ER cholesterol content is not a primary regulator of SREBP-1 cleavage, as its activity poorly correlates with cholesterol metabolism. Cell experiments indicate that processing of SREBP-1, unlike SREBP-2, is not fully suppressed in the presence of excess cholesterol. Moreover, only SREBP-1 proteolytic activation is enhanced by insulin or fasted/refed conditions [7], [8], but this is suppressed by polyunsaturated fatty acids or AMP-activated protein kinase [9], [10]. However, despite increasing evidence for the difference between the SREBP-1 and SREBP-2 processing [11], [12], the molecular mechanism underlying the SREBP-1-specific regulation remains unclear.

In this study, we first found that in WATs of plin−/− mice, the amount of nuclear SREBP-1, but not SREBP-2, was greatly reduced as was TAG accumulation. Thus, we focused on the interplay between the activation of SREBP-1 and LD generation in differentiated adipocytes. During adipogenesis, lipogenic gene expression is augmented under the control of SREBP-1, and the number of LDs rich in TAG grows simultaneously with increased plin expression. However, how SREBP-1 is proteolytically activated to enhance its target gene expression concurrently with LD biogenesis is unclear. We describe a novel interplay between LD formation and SREBP-1 proteolytic activation in adipocytes.

## Methods

### Materials

Thapsigargin, tunicamycin, insulin, 5α-cholestane, a protease inhibitor cocktail and dexamethazone were purchased from Sigma. 4-(2-aminoethyl) benzenesulfonyl fluoride hydrochloride (AEBSF), 3-isobutyl-1-methylxanthine, and pioglitazone were obtained from Wako. *N*-acetyl-Leu-Leu-norleucinal (ALLN) was from Nacalai Tesque. 19-hydroxycholesterol was purchased from Toronto Research Chemicals Inc.

### Cultured Cells Primary

MEFs were isolated from C57BL/6J mice embryos at 13.5 days post coitum by trypsinization [13]. All experiments were performed at passage 2. 3T3-L1 preadipocytes and MEFs were differentiated as described previously [14], [15]. PPARγ-expressing 3T3-L1 cells were cultured in D'MEM with 10% calf serum, 100 units/mL penicillin and 100 µg/mL streptomycin, and differentiated into adipocytes without any hormonal stimulation. PPARγ-expressing MEFs were maintained in D'MEM with 10% FBS, 100 units/mL penicillin and 100 µg/mL streptomycin, and differentiated into adipocytes without any hormonal stimulation.

### Antibodies

Anti-β-actin, anti-FLAG (M2) and anti-G58K antibodies were obtained from Sigma. Anti-SREBP-1 (2A4) and anti-PPARγ antibodies were purchased from Santa Cruz. Anti-Akt and Anti-phosphorylated Akt (Ser473, Thr308) were from Cell Signaling, anti-perilipin antibody from PROGEN, and anti-GFP and anti-protein disulfide isomerase (PDI) antibodies from Abcom. The anti-SREBP-2 polyclonal antibody has been described previously [16].

### Plasmids

A lentiviral expression plasmid for Flag-tagged PPARγ2 was described previously [15]. A retroviral expression plasmid for Flag-tagged plin was similarly constructed by inserting a PCR fragment encoding mouse plin into CSII-EF-MCS-IRES2-Venus (RIKEN). A retroviral expression plasmid for the GFP-SREBP-1 fusion protein was constructed by inserting a PCR fragment [GFP followed by a nuclear localization signal including a Lys-Lys-Lys-Arg-Lys motif plus the C-terminal half of human SREBP-1a (amino acids 392-1134)] into pMX [15].

### Mice and Tissues

Wild-type and plin−/− mice on C57BL/6 background were obtained from Jackson Laboratory. Epididymal and subcutaneous fat were harvested from plin−/−, +/− or −/− mice. The Institutional Animal Care and Research Advisory Committee at the University of Tokyo approved all animal procedures.

### Real-time

*PCR* Total cell RNA was extracted and reverse transcribed with Superscript III (Invitrogen). Fluorescence real-time PCR was performed on a StepOnePlus system using TaqMan Gene Expression Assays (Applied Biosystems). S17 rRNA protein transcript was used as an internal control to normalize variations in RNA amounts.

### Subcellular Fractionation

Cells were homogenized on ice in buffer A [10 mM HEPES-NaOH (pH 7.4), 250 mM sucrose, 1 mM EDTA, and a protease inhibitor cocktail with 50 µM ALLN] using 30 strokes of a 1 mL syringe with 25G needle. Cell homogenates were centrifuged at 12,500×g for 10 min to remove larger organelles. Supernatants were centrifuged at 100,000×g for 30 min to obtain the cytosol (supernatant) and microsome fractions (pellet). For ER isolation, the microsome fractions were resuspended with buffer A and applied on 0–25% OptiPrep density gradient media (Axis-shield, Norway). After centrifugation at 100,000×g for 3 h, 20 fractions were collected. ER fractions were determined by immunoblotting using antibodies against PDI. To obtain ER fractions from subcutaneous fats of plin−/− or +/+ mice, fats were homogenized in buffer A using a TissueRuptor (Qiagen), and homogenates were centrifuged at 1,000×g for 10 min. Supernatants were further fractionated as described above.

### Free Cholesterol Quantification Analysis

Whole cell lysates or pooled ER fractions after ultracentrifugation were mixed with an equal amount of chloroform/methanol (2∶1; v/v). After vortexing for 15 min and resting for 10 min, the samples were centrifuged for 15 min. Organic layers were collected and vacuum dried. The pellets were resolved using isopropanol. Free cholesterol amounts were quantified by the Free Cholesterol C-test Wako (Wako).

### Free Cholesterol Quantification Analysis by GC-MS

After pooled ER fractions from subcutaneous fats were mixed with 19-hydroxycholesterol (an internal control), total lipids were extracted as described above. Free cholesterol was converted into trimethylsilyl ethers in a mixture of trimethylchlorosilane, 1,1,1,3,3,3-hexamethyldisilazane, and dried pyridine (1∶3∶9, v:v:v) for 30 min at room temperature using 5α-cholestane as an internal standard. Trimethylsilyl ethers of free cholesterol were quantified with a Shimadzu GCMS-QP2010 system (Shimadzu, Kyoto, Japan) consisting of a model GC-2010 gas chromatograph connected to a mode QP2010 electron-impact (EI) mass spectrometer equipped with an SPB-1 fused silica capillary column (60 m ×0.25 mm, 0.25 mm phase thickness; Supelco Inc., Bellefonte, PA, USA). In the oven temperature program, the temperature was initiated at 180 °C for 1 min and then raised to 250 °C at 20°C/min and to 290°C at 5°C/min and held for 45 min. The injector and detector temperatures were at 300°C. Monitored ions were determined as previously described [17]. Using 19-hydroxycholesterol and 5α-cholestane as internal standards, ER cholesterol amounts were quantified.

### Retrovirus or Lentivirus Infection, Oil Red O Staining and TAG Quantification Analysis

Methods were as previously described [15].

### Statistical Analysis

Results are presented as mean ± standard deviation (±SD) and evaluated by Student’s *t*-test for 2 groups. Significance was assumed at p<0.05 and <0.01.

## Results

### Plin Deficiency Suppresses SREBP-1 Activation in WATs

**Figure 1:**
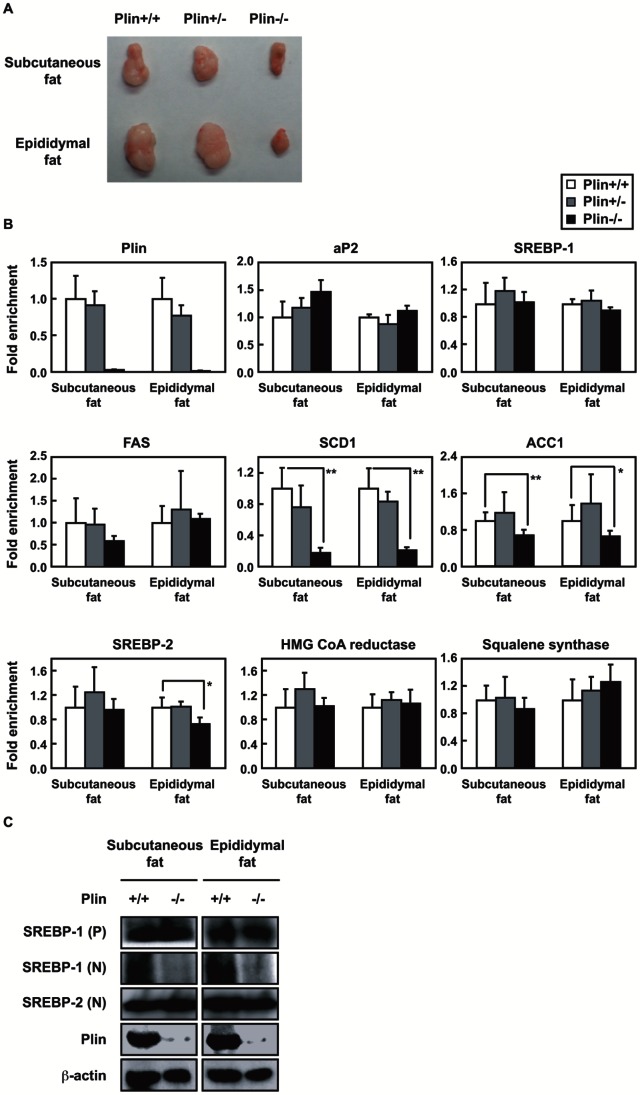
Gene and protein expression in plin+/+, +/− and −/− mice adipose tissues. (**A**) mice WATs. (**B**) Total RNA was extracted and reverse transcribed from tissues. Quantitative RT-PCR was performed using Taq-Man Gene Expression Assays. S17 rRNA was used as an internal control to normalize the mRNA levels. Data are means ±SD of 5 or 6 mice. The relative mRNA level of each gene in plin+/+ mice is taken as 1.0. *p<0.05 and **p<0.01 using unpaired Student’s *t* test. (**C**) Proteins were extracted from tissues and immunoblots were performed. *P* and *N* denote the precursor and nuclear form of SREBPs, respectively.

Previous histological analyses have revealed that LDs in WATs are smaller in plin−/− mice than in wild-type animals [18]. Subcutaneous or epididymal fat size reduced significantly (Figure 1A). This was thought to be due to continuously induced basal lipolysis in the absence of plin. In this study, we investigated the expression of genes related to adipogenesis and lipid metabolism in the adipose tissues of plin−/−, +/− and +/+ mice. The plin gene expression in the tissues of plin+/+ and +/− mice did not differ much, and the mRNA levels of other genes were unaltered in both animals. (Figure 1B). In contrast, compared with plin+/+ mice, the expression of SREBP-1 target genes (SCD1; stearoyl CoA desaturase-1, and ACC1; acetyl CoA carboxylase-1) was significantly reduced in both adipose tissues of plin−/− mice with no alteration of adipocyte marker genes aP2 and SREBP-1. The mRNA levels of squalene synthase and HMG CoA reductase, thought to be SREBP-2 targets, were not altered for the 3 genotypes despite a mild decrease in SREBP-2 mRNA levels in the epididymal fat of plin−/− mice. Immunoblots showed that the amount of the active nuclear SREBP-1 protein in these tissues was greatly reduced in plin−/− than in wild-type mice (Figure 1C). No difference was seen in the amount of nuclear SREBP-2 protein [Figure 1C, SREBP-1(N) and -2(N)]. These results suggest that in adipose tissues of plin−/− mice the reduced SREBP-1 activation with the suppressed gene expression of its targets decreases lipid accumulation by lowering fatty acid and TAG synthesis, probably with induced basal lipolysis due to plin deficiency. A deficiency of plin causes a considerable reduction in active SREBP-1, and SREBP-1, rather than SREBP-2, in cooperation with plin, may play a critical role in lipid accumulation in adipocytes.

### SREBP-1 Activation is Suppressed in Differentiated MEFs Prepared from plin−/− Mice

**Figure 2:**
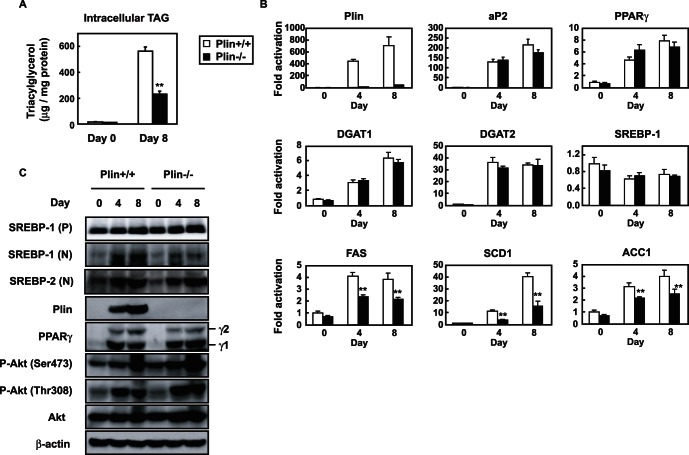
Plin deficiency suppresses TAG accumulation and SREBP-1 activation in differentiated MEFs. (**A**) MEFs prepared form plin+/+ or −/− mice (n = 3) were differentiated for 8 days. The amount of intracellular TAG was determined on days 0 and 8. Data are means ±SD; **p<0.01 *versus* plin+/+. (**B**) Quantitative RT-PCR analyses showing the gene expression patterns in differentiating plin+/+ or −/− MEFs (n = 3). S17 rRNA was used as an internal control to normalize the mRNA level of each gene. Data are means ±SD; the levels in plin+/+ on day 0 are taken as 1.0. **p<0.01 *versus* plin+/+. (**C**) Immunoblots showing the levels of proteins during adipogenesis of plin+/+ or −/− MEFs. *P* and *N* denote the precursor and nuclear form of SREBPs, respectively.

Because SREBP-1 was also discovered as an adipogenic regulatory factor [5], we investigated the effect of plin deficiency on adipogenesis. MEFs prepared from plin+/+ or −/− mice were differentiated into adipocytes to compare the transcriptional changes and TAG accumulation during adipogenesis. As in *in vivo* findings, TAG accumulation (day 8; Figure 2A) was greatly reduced in the plin−/− differentiated adipocytes (60% reduction). Plin deficiency did not affect the expression of genes induced during adipogenesis such as PPARγ (master regulator of adipogenesis), its target aP2, and diacylglycerol acyltransferases 1 and 2 (DGAT1 and 2, essential for TAG biosynthesis). This suggests that adipogenesis progressed normally (Figure 2B). Unlike 3T3-L1 cells, MEFs maintained a constant SREBP-1 mRNA level during adipogenesis. Conversely, mRNA of SREBP-1 targets (FAS; fatty acid synthase, SCD1 and ACC1) notably declined in the absence of plin. Immunoblots revealed that active nuclear SREBP-1 (N) increased with advancing adipogenesis in both types of MEF, but the degree of SREBP-1 activation in the plin-null cells was notably lower, without any difference in the PPARγ protein (γ1 and γ2) and nuclear SREBP-2 (N) levels in the groups (Figure 2C). As with *in vivo* findings in plin−/− adipose tissues, SREBP-2 was efficiently activated in differentiating plin−/− MEFs as in plin+/+ MEFs. When 3T3-L1, MEFs and primary preadipocytes were differentiated into adipocytes, SREBP-1 and SREBP-2 nuclear forms dramatically increased concomitantly with the emergence of plin during adipogenesis (Figure s1). This suggests that sufficient SREBP-1 activation requires further LD generation aided by plin. This was also observed in adipocytes differentiated from primary preadipocytes prepared from plin+/− and −/− animals (Figure s2). Insulin signaling is involved in SREBP-1 proteolytic activation [19]; Akt activation was examined in these cells to confirm if plin depletion diminished this pathway. Immunoblots show that plin deficiency had no effect on activation of insulin signaling during adipocyte differentiation (Figure 2C).

### Enforced Plin Expression in Differentiating Plin−/− MEFs Promotes TAG Accumulation and SREBP-1 Activation Followed by an Increase in its Target Gene Expression

**Figure 3:**
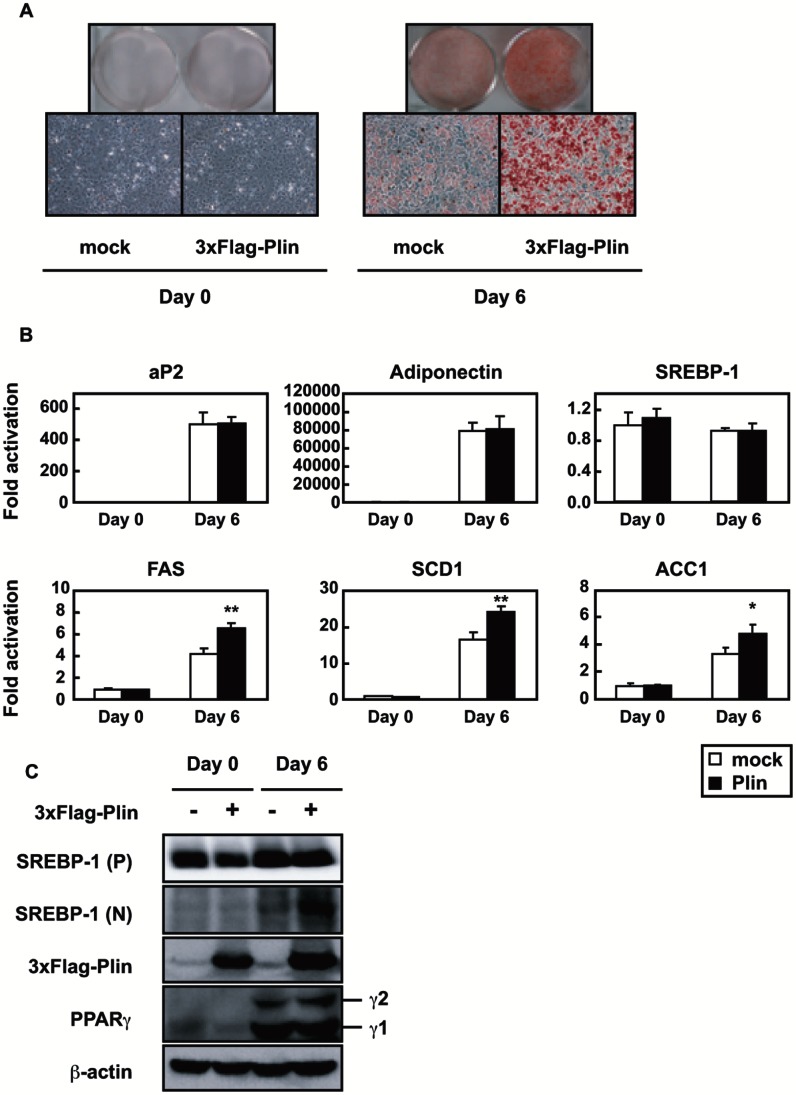
Enforced expression of plin in plin−/− MEFs promotes LD generation and SREBP-1 activation. (**A**) Pictures of Oil Red O staining of MEFs infected with the mock or 3xFlag-plin virus. Staining was performed on days 0 and 6. (**B**) Quantitative RT-PCR analyses showing the gene expression patterns in mock or plin expressing MEFs (n = 3). S17 rRNA was used as an internal control to normalize the mRNA level of each gene. Data are means ±SD; the levels in mock cells on day 0 are taken as 1.0. *p<0.05 and **p<0.01 *versus* mock. (**C**) Immunoblots showing the levels of proteins in mock or plin expressing MEFs during adipogenesis. *P* and *N* denote the precursor and nuclear form of SREBP-1, respectively.

To confirm LD formation and SREBP-1 activation interplay, plin−/− MEFs were infected with a flag-tagged plin expression retrovirus and differentiated into adipocytes. Plin expression greatly stimulated lipid accumulation only in differentiated adipocytes (Figure 3A; day 6). Gene expression of PPARγ targets (aP2 and adiponectin) and SREBP-1 was not affected by plin expression on days 0 and 6 (Figure 3B). Immunoblots demonstrated that the plin protein expression had no effect on the PPARγ protein levels (γ1 and γ2) on day 6 (Figure 3C), indicating that differentiation progressed almost equally in these MEFs in the presence or absence of flag-tagged plin. Conversely, SREBP-1 target mRNA levels were substantially elevated in the presence of flag-tagged plin on day 6 (Figure 3B) with increased active SREBP-1 (N) (Figure 3C). It is noteworthy that nuclear SREBP-1 (N) increased in response to plin-mediated LD generation. From these *in vivo* and *in vitro* findings, in plin−/− adipose tissues, it seems likely that plin deficiency attenuated LD formation, suppressing SREBP-1 activation and its target gene expression, further leading to declining TAG accumulation and LD biogenesis.

### Increased LD Formation Facilitates SREBP-1 Activation

**Figure 4:**
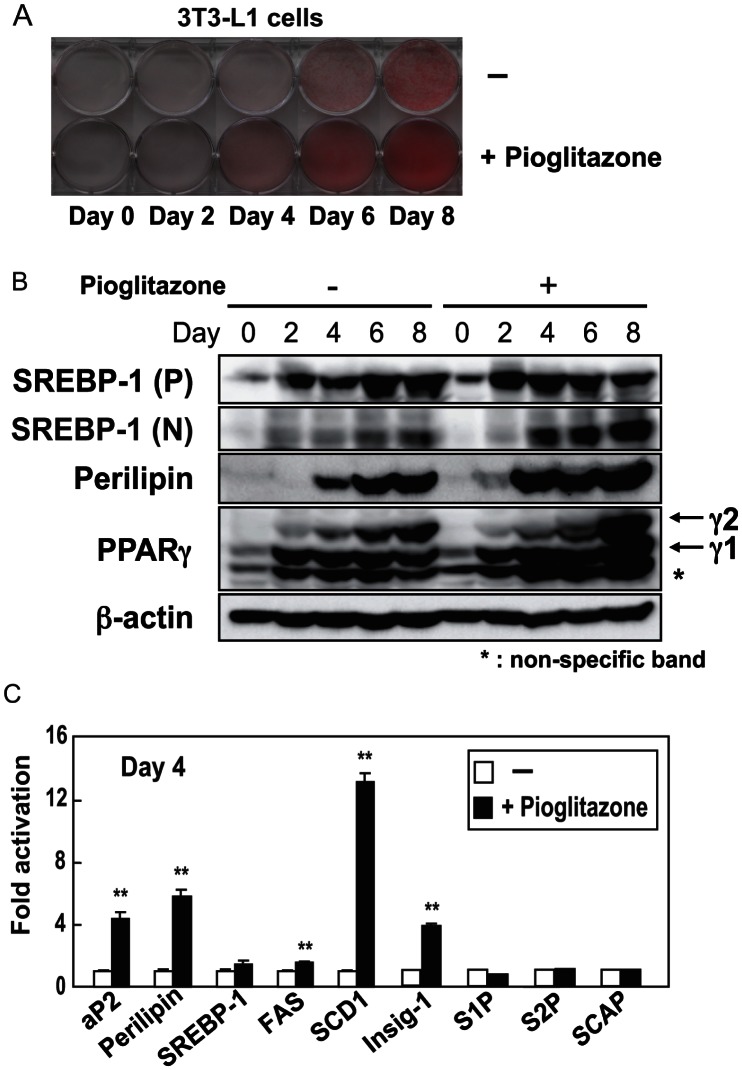
SREBP-1 processing is enhanced in differentiating 3T3-L1 cells cultured with the synthetic PPARγ agonist pioglitazone. (**A**) 3T3-L1 cells were induced to differentiate with or without pioglitazone (10 µM) for initial 2 days. Pictures of Oil Red O staining of differentiating 3T3-L1 cells. (**B**) Immunoblots showing the levels of proteins in cells differentiating with or without pioglitazone. *P* and *N* denote the precursor and nuclear form of SREBP-1, respectively. (**C**) Quantitative RT-PCR analyses showing the gene expression patterns in cells differentiating (day 4) with or without pioglitazone. S17 rRNA was used as an internal control to normalize the mRNA level of each gene. Data are means ±SD (n = 3). **p<0.01 *versus* without pioglitazone.

To examine if stimulated plin expression in preadipocytes accelerates TAG accumulation and SREBP-1 activation, 3T3-L1 cells were differentiated with the synthetic PPARγ agonist pioglitazone to induce plin expression, a PPARγ target [20]. Pioglitazone treatment caused a significant rise in plin protein and TAG accumulation on day 4 or later (Figure 4A). Immunoblots revealed that nuclear SREBP-1 (N) increased greatly on days 4–8 in the agonist-treated cells (Figure 4B). In differentiating adipocytes, the SREBP-1 target mRNA levels (FAS, SCD1 and Insig-1) were elevated in the presence of the agonist (Figure 4C), suggesting that PPARγ-induced LD formation, due to excess production of plin, leads to increased SREBP-1 activation and expression of its target gene. Conversely, no changes were seen in genes (SCAP, S1P and S2P) thought to create a favorable environment for SREBP-1 processing under certain conditions (Figure 4C).

**Figure 5:**
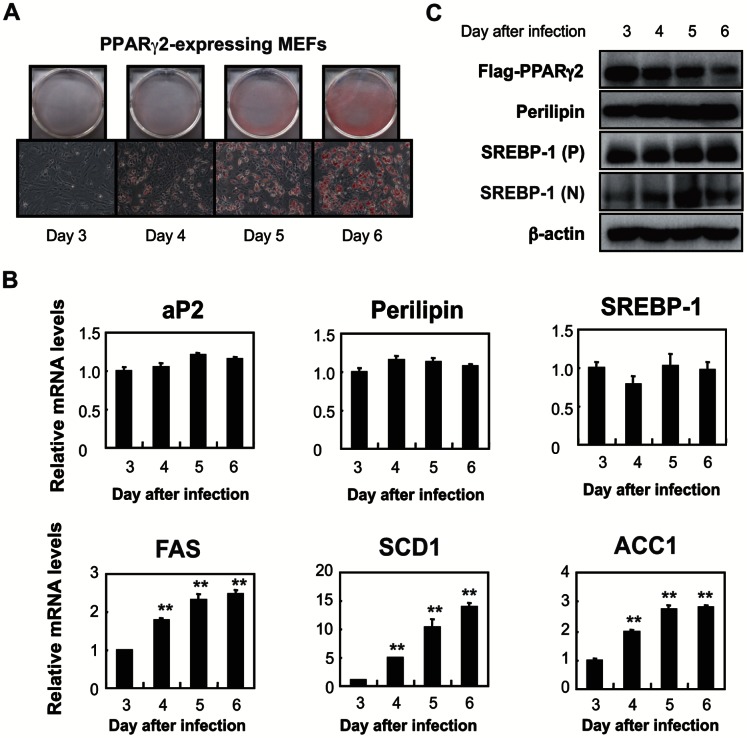
TAG accumulation promotes SREBP-1 activation and its target gene expression in PPAR γ2-expressing MEFs. (**A**) Pictures of Oil Red O staining of PPARγ2-expressing MEFs 3-6 days after infection with a flag-tagged PPARγ2 expression virus. (**B**) Quantitative RT-PCR analyses showing the gene expression patterns in differentiating MEFs on days 3-6 (n = 3). S17 rRNA was used as an internal control to normalize the mRNA level of each gene. Data are means ±SD; the relative mRNA levels on day 3 are taken as 1.0. ** p<0.01 *versus* day 3. (**C**) Immunoblots showing the levels of proteins in PPARγ-expressing MEFs. *P* and *N* denote the precursor and nuclear form of SREBP-1, respectively.

In another study we used MEFs infected with a flag-tagged PPARγ2 expression lentivirus. These cells spontaneously differentiate into adipocytes without a PPARγ agonist or a mixture of insulin, dexamethazone, and 3-isobuthyl-1-methylxanthine, which is normally required for 3T3-L1 adipocyte differentiation. This allowed us to ignore hormonal or drug effects on LD formation and SREBP-1 activation. After 3 days of infection, PPARγ-MEFs began to accumulate LDs, and the number of LDs stained by Oil Red O increased for the subsequent 3 days (Figure 5A). Expression of PPARγ targets (aP2 and Plin) was augmented on day 3 and remained constant for 4 days (Figure 5B). The SREBP-1 mRNA levels were unchanged for these days; SREBP-1 targets increased substantially. Immunoblots show that nuclear SREBP-1 abundance increased from day 3 to 5 despite no marked change in SREBP-1 precursor (Figure 5C). On day 6, the flag-tagged PPARγ2 protein decreased, but plin protein levels were almost constant for 4 days. These results indicate that sufficient LD formation with plin production enables SREBP-1 activation in differentiating adipocytes.

### ER Membrane Cholesterol Content is Reduced after LD Formation and SREBP-1 is Processed by an S1P-dependent Pathway

**Figure 6:**
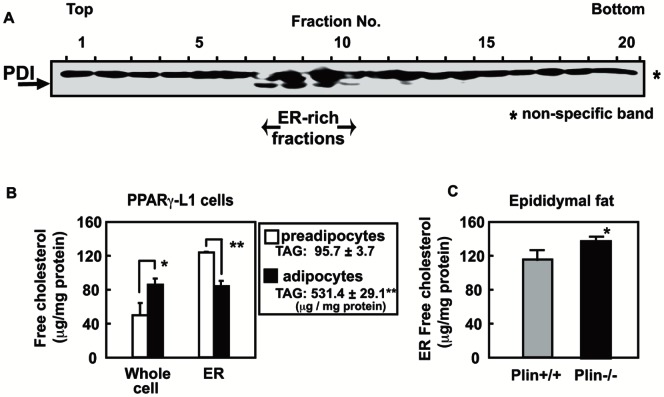
Adipocyte differentiation produces changes in the ER cholesterol content. (**A**) Differentiated PPARγ-expressing 3T3-L1 cells (day 11 after infection) were fractionated by ultracentrifugation. Equal volumes of each fraction were subjected to immunoblot for the ER (PDI) marker. (**B**) Free cholesterol amounts in whole cell lysates or ER fractions of PPARγ-expressing 3T3-L1 cells were determined (n = 3). *p<0.05 and **p<0.01 *versus* preadipocytes. (**C**) Free cholesterol amounts in ER fractions prepared from epididymal fats of plin+/+ and −/− mice were determined. *p<0.05 *versus* plin+/+.

The prevailing model indicates that TAG is synthesized and stored between the leaflets of the ER bilayer membrane; LDs are thought to be released into the cytoplasm by budding from the outer leaflet of the ER membrane. We assumed that the ER membrane microenvironment should be altered concurrently with LD formation. To assess this, the ER membrane free cholesterol content was determined in 3T3-L1 cells infected with a flag-tagged PPARγ2 expression lentivirus and spontaneously differentiated into adipocytes. Protein disulfide isomerase-rich microsomal fractions were isolated by ultracentrifugation (Figure 6A). The free cholesterol content in these ER-rich fractions was substantially lowered in PPARγ-L1 cells on day 11 (differentiated adipocytes) than preadipocytes on day 5 after infection (Figure 6B). The intracellular TAG level was much higher in differentiated adipocytes than preadipocytes. LD generation decreases the ER free cholesterol content, which may promote SREBP processing in the vicinity of the ER and Golgi, to increase the nuclear form of SREBP-1 and SREBP-2 as observed in fats and MEFs of plin+/+ mice (Figures 1C and 2C). Indeed, when the ER-rich fractions were prepared from epididymal fats of plin+/+ or −/− mice, the free cholesterol level in plin+/+ mice was significantly lower than that in plin−/− mice (Figure 6C). Since epididymal fats contained several types of cells other than mature adipocytes, the difference in the ER cholesterol level was relatively smaller than expected from that in PPARγ-L1 cells (Figure 6B). These results indicate an inverse relationship between the ER cholesterol content and LD formation.

**Figure 7:**
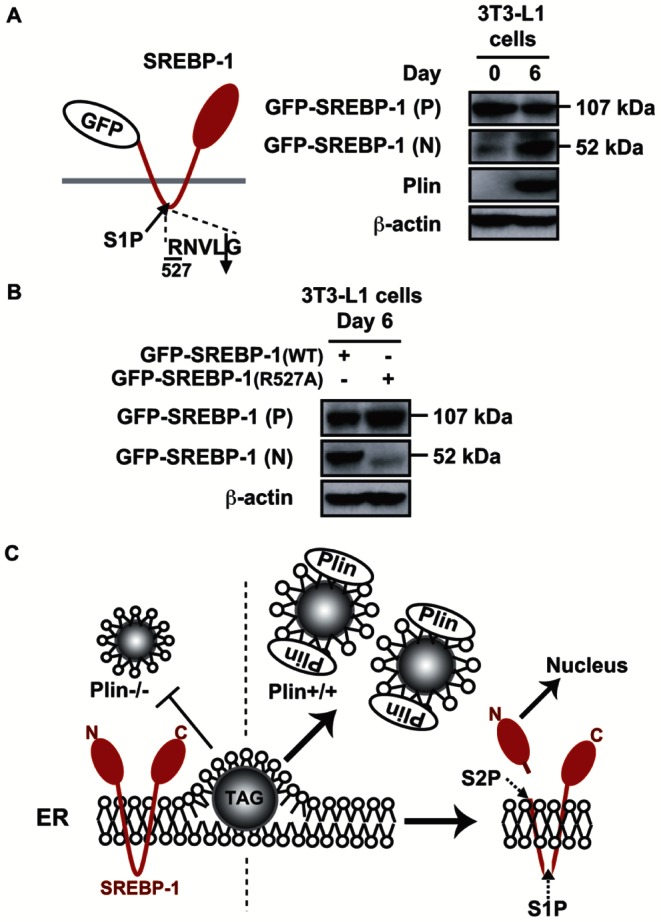
SREBP-1 is processed in an S1P-dependent manner in differentiated adipocytes. (**A**) 3T3-L1 cells were infected with an expression retrovirus for the GFP-SREBP-1 fusion protein and then differentiated into adipocytes. Immunoblots showing the levels of GFP-containing proteins on days 0 and 6. *P* and *N* denote the precursor and nuclear form of the GFP-SREBP-1 fusion protein, respectively. (**B**) Immunoblots showing protein levels in differentiated 3T3-L1 cells expressing GFP-SREBP-1(wild type) or GFP-SREBP-1(R527A) with a mutated S1P cleavage site. *P* and *N* denote the precursor and nuclear form of the GFP-SREBP-1 fusion protein, respectively. (**C**) A model showing plin-mediated LD formation triggers SREBP-1 activation through the canonical pathway controlled by two cleavage enzymes S1P and S2P in the Golgi compartment. The cleaved form of SREBP-1 transported to the nucleus stimulates its target gene expression and subsequent TAG synthesis, which seems likely to further enhance LD formation, creating a positive feedback loop.

To examine if SREBP-1 was processed through the canonical pathway controlled by two cleavage enzymes S1P and S2P that reside in the Golgi compartment, but not through the caspase-dependent pathway that is cholesterol insensitive [21], 3 lines of adipocytes were treated with the serine protease inhibitor AEBSF, which had shown to hinder S1P activity and the cleavage of SREBP-2 and ATF6, another S1P/S2P substrate [22]. Treatment of the cells (3T3-L1 cells, day 7; MEFs, day 5; and PPARγ-L1 cells, day8) with AEBSF for 9 h resulted in a robust reduction in nuclear SREBP-1 (N) without any effect on the precursor form (P) or plin expression (Figure s3A). Longer treatment (24 h) reduced the number of cells stained by Oil Red O in all groups, suggesting that the rapid decrease in active SREBP-1 affected intracellular TAG accumulation and LD formation (Figure s3B). To further validate the involvement of S1P in induced SREBP-1 processing, we examined the processing of a mutant form of SREBP-1, which lacks the S1P cleavage site, in differentiated 3T3-L1 cells. We generated a retroviral expression construct for the GFP-SREBP-1 fusion protein, which lacks the transcription factor domain but contains 2 transmembrane domains and the C-terminal portion, to eliminate transcriptional functions (Figure 7A). The GFP-SREBP-1 fusion protein [GFP-SREBP-1 (P), 107 kDa] was poorly processed in preadipocytes but efficiently cleaved into a smaller-sized fragment [GFP-SREBP-1 (N), 52 kDa] recognized by the anti-GFP antibody in differentiated 3t3-L1 cells (day 6). Once the S1P cleavage site (Figure 7A) localized in the ER luminal loop between 2 transmembrane domains was mutated (R527A) [23], the mutant protein [GFP-SREBP-1(R527A)] was scarcely cleaved (Figure 7B), in agreement with the results using AEBSF (Figure s3A). Thus, SREBP-1 is thought to be processed in the vicinity of the Golgi compartment through the S1P-dependent pathway in differentiating adipocytes but is not directly cleaved by unknown proteases (Figure 7C).

## Discussion

When 3T3-L1, MEFs, and primary preadipocytes were differentiated into adipocytes, the nuclear forms of both SREBP-1 and SREBP-2 increased dramatically with plin emergence during adipogenesis (Figure s1). In contrast, in plin−/− adipose tissues and differentiated MEFs, decreased LDs due to the lack of a critical LD surface protein resulted in reduced nuclear SREBP-1, but not SREBP-2 (Figure 1 and 2). Increased nuclear SREBPs during adipogenesis could be due to stabilization of those rapidly degraded by the ubiquitin-proteasome pathway [24], enhancement of direct cleavage of precursor forms localized in the ER membrane without two-step processing in the Golgi [21], or aberrant alternative splicing of SREBP-1 [25]. The finding that the nuclear SREBPs in 3T3-L1 adipocytes were substantially stabilized in the presence of proteasome inhibitors and highly unstable negates the first possibility (data not shown). We ruled out the second alternative because a serine protease inhibitor AEBSF, which inactivates S1P, strongly hindered SREBP-1 processing and a fusion SREBP-1 protein ectopically expressed in adipocytes, unlike a mutant protein lacking the S1P cleavage site, was efficiently cleaved (Figure 7). Reverse transcription PCR experiments using several types of primer pair revealed no occurrence of an aberrant alternative splicing producing an active nuclear form of SREBP-1 during adipogenesis (data not shown). These results indicate that SREBP-1 is activated through the S1P-mediated proteolytic pathway in response to plin-induced LD generation in differentiating adipocytes.

We have shown that LD generation, induced by an ample supply of plin, accelerates SREBP-1 proteolytic processing during adipogenesis. In contrast, a recent study demonstrated that adipocytes with deletions of both DGAT-1 and -2 severely lacked TAG and did not have LDs. This indicates that DGATs account for nearly all TAG synthesis in adipocytes and appear to be required for LD formation during adipogenesis [26]. In plin−/− differentiated MEFs gene expression of DGATs was unaltered, but the mRNA levels of genes for fatty acid synthesis were reduced, leading to decreased TAG accumulation (Figure 2). Under these conditions, suppression of SREBP-1 activation resulting from poor proteolytic processing may be a major cause of impaired TAG synthesis and accumulation. Previous microarray analyses of the gene expression profile of WAT of plin−/− and +/+ mice revealed a coordinated downregulation of genes involved in lipid biosynthesis, notably a significant decrease in SCD-1 mRNA [27], consistent with our findings (Figure 1). Suppression of SREBP-1 activation due to insufficient LD formation accounts for a pronounced decline in SCD-1 gene expression that is largely regulated by SREBP-1. Since in plin−/− adipocytes, small LDs surrounded by ADRP, another LD surface protein, are thought to be exposed to continuously induced basal lipolysis [18], decreased LDs and TAG accumulation in plin−/− differentiated MEFs cannot be accounted for only by impaired TAG synthesis. However, when SREBP-1 gene expression in differentiated 3T3-L1 adipocytes was reduced by half with a lentivirus vector expressing SREBP-1 shRNA, a substantial decline in the number of cells stained by Oil Red O was observed (Figure s4A). SREBP-1 shRNA diminished the active SREBP-1 (N) protein level on day 6 by about 50%, but had no effect on the expression of SREBP-2, aP2, and plin, with a substantial decrease in the SCD1 mRNA level (Figure s4B and s4C). These results indicate that SREBP-1 activation correlates well with LD formation in concert with increased *de novo* TAG synthesis. During adipogenesis plin-mediated LD biogenesis accelerates SREBP-1 processing and stimulates SREBP-1 target gene expression and subsequent TAG synthesis, which further enhances LD formation, creating a positive feedback loop (Figure 7C).

SREBP-1 and -2 share a common structure with 2 transmembrane domains and are activated through the same proteolytic pathway. SREBP-2 processing is tightly controlled by the cholesterol level in the ER membrane [28], whereas the SREBP-1 processing is accelerated in the postprandial state after fasting in response to insulin secretion, and is also regulated by the cholesterol level in most cultured cells [7], [8], [29]. We have shown that LD generation, induced by an ample supply of plin, accelerates SREBP-1 proteolytic processing during adipogenesis, whereas in the absence of plin SREBP-1 processing is severely impaired along with decreased LD formation despite no alteration of SREBP-2 processing (Figures 1 and 2). The ER cholesterol content reduced during adipogenesis (Figure 6), consistent with a previous report [30], suggesting that this decrease should facilitate the SREBP-2 proteolytic activation in adipocytes. Because the LD phospholipid monolayer is derived from the outer leaflet of the ER membrane bilayer, this decline seems likely after LD generation. Indeed, LDs are surrounded by cholesterol-rich ER-like surface layer structures [31]. In contrast, the nuclear form of SREBP-1, but not SREBP-2, was decreased in plin−/− adipose tissues and differentiated MEFs; this decrease was restored by enforced plin expression. Thus, the sufficient SREBP-1 cleavage along with plin-mediated LD generation is caused by something other than the decreased ER cholesterol level. At this moment precise mechanism by which active LD generation triggers the SREBP-1 proteolytic activation is unknown. We excluded the possibility that impaired insulin signaling pathway accounts for reduced SREBP-1 processing in plin−/− adipocytes because of no alteration in Akt phosphorylation (Figure 2C). When differentiated PPARγ-L1 adipocytes were treated with a phosphoinositide 3-kinase inhibitor (LY294,002 or wortmannin) for 24 h, SREBP-1 processing was unaffected despite substantial suppression of Akt phosphorylation at serine 473 (data not shown). Another plausible scenario is that LD formation leads to the disappearance of Insigs interacting with the SREBP-SCAP complex in ER and hamper SREBP processing. When Insig-1 and -2 were depleted in the ER membrane of double knockout mice, SREBP processing was stimulated in particularly SREBP-1 [32]. Although we identified whole LD surface proteins by mass spectrometry analyses to unveil an exit of Insigs from ER to LDs, Insigs were not detected in LDs despite identification of several ER proteins (data not shown). Moreover, immunoblots using Insig-1 antibody revealed no significant changes in the protein in the ER membrane fraction (data not shown). Alternatively, the balance of membrane phosphatidylcholine (PC)/phosphatidylethanolamine ratio may be changed in response to plin-mediated LD formation, thereby affecting protein trafficking from the ER to the Golgi. Recent reports demonstrated that blocking of PC promotes elevated levels of nuclear SREBP-1, but not SREBP-2, resulting in increased SREBP-1-dependent gene expression, and in elevated lipogenesis and LD formation [33], [34]. Further studies are required to elucidate the exact mechanism underlying SREBP-1 proteolytic activation probably promoted by alterations in the ER membrane microenvironment in concert with LD formation.

In conclusion, studies using plin−/− mice confirmed that attenuated LD formation caused by the lack of plin resulted in decreased SREBP-1 activation, resulting in reduced lipogenic gene expression. Upon formation of LD that largely derived from the ER, the altered ER microenvironment in adipocytes may facilitate SREBP-1 proteolytic processing controlled by two cleavage enzymes S1P and S2P that reside in the Golgi compartment. Overall, these results reveal the novel interplay between plin-mediated LD formation and SREBP-1 activation through a positive feedback loop during adipogenesis.

## Supporting Information

Figure S1 **Protein expression in three types of differentiating cells.** Immunoblots showing the levels of proteins in differentiating 3T3-L1 cells, MEFs and fibroblastic primary preadipocytes. *P* and *N* denote the precursor and nuclear form of SREBPs, respectively.
(TIFF)

Figure S2 **Plin deficiency suppresses TAG accumulation and SREBP-1 activation in differentiated primary adipocytes.** (**A**) PCR analyses for genotyping. (**B**) Pictures of Oil Red O staining of differentiated primary adipocytes prepared from three types of mice. (**C**) Immunoblots showing the levels of proteins during adipogenesis of plin+/− or −/− MEFs. *N* denotes the nuclear form of SREBP-1. (**D**) Quantitative RT-PCR analyses showing the gene expression patterns in the differentiating Plin+/− or −/− MEFs. S17 rRNA was used as an internal control to normalize the mRNA level of each gene. Data are means ± SD (n = 3).
(TIFF)

Figure S3 **AEBSF treatment reduces intracellular TAG accumulation in differentiated cells.** (**A**) Differentiated 3T3-L1 (day 7), MEFs (day 5) and PPARγ-expressing 3T3-L1 cells (day 8 after infection) were treated with or without 300 µM AEBSF for 24 h. Immunoblots showing the levels of proteins in three differentiated cells. *P* and *N* denote the precursor and nuclear form of SREBP-1, respectively. (**B**) Pictures of Oil Red O staining of these cells after the treatment.
(TIFF)

Figure S4 **Reduced SREBP-1 expression decreases TAG accumulation along with its target gene expression in differentiated 3T3-L1 cells.** 3T3-L1 cells were infected with one of lentiviral vectors expressing control shRNA or shRNA for SREBP-1 on day -1. On day 0 the cells were induced to differentiate. The lentiviral plasmid for shRNA of mouse SREBP-1 was constructed by recombining pCS-RfA-EG (RIKEN) with the pENTR4-H1 (RIKEN) inserted by oligonucleotide DNA for shRNA expression [14]. The target sequences are as follows: SREBP-1; 5′-GCGGCTGTTGTCTACCATAAG-3′, control (Scramble II Duplex from Dharmacon); 5′- GCGCGCTTTGTAGGATTCG –3′. (A) Pictures of Oil Red O staining of differentiated 3T3-L1 cells infected with one of shRNA lentivirus vectors on day 6. (B) Immunoblots showing the levels of proteins in differentiated 3T3-L1 cells. *P* and *N* denote the precursor and nuclear form of SREBP-1, respectively. (C) Quantitative RT-PCR analyses showing the gene expression patterns in differentiating 3T3-L1 cells on day 0 and 6 (n = 3). S17 rRNA was used as an internal control to normalize the mRNA level of each gene. The relative mRNA levels in the cells infected with the control virus on day 0 are considered as 1.0. **p<0.01 *versus* sh control.
(TIFF)
