# Supplementary figures and images for: Perilipin-Mediated Lipid Droplet Formation in Adipocytes Promotes Sterol Regulatory Element-Binding Protein-1 Processing and Triacylglyceride Accumulation
Source: PLoS One. 2013 May 29;8(5):e64605. doi: 10.1371/journal.pone.0064605 (PMC3667186; doi:10.1371/journal.pone.0064605)

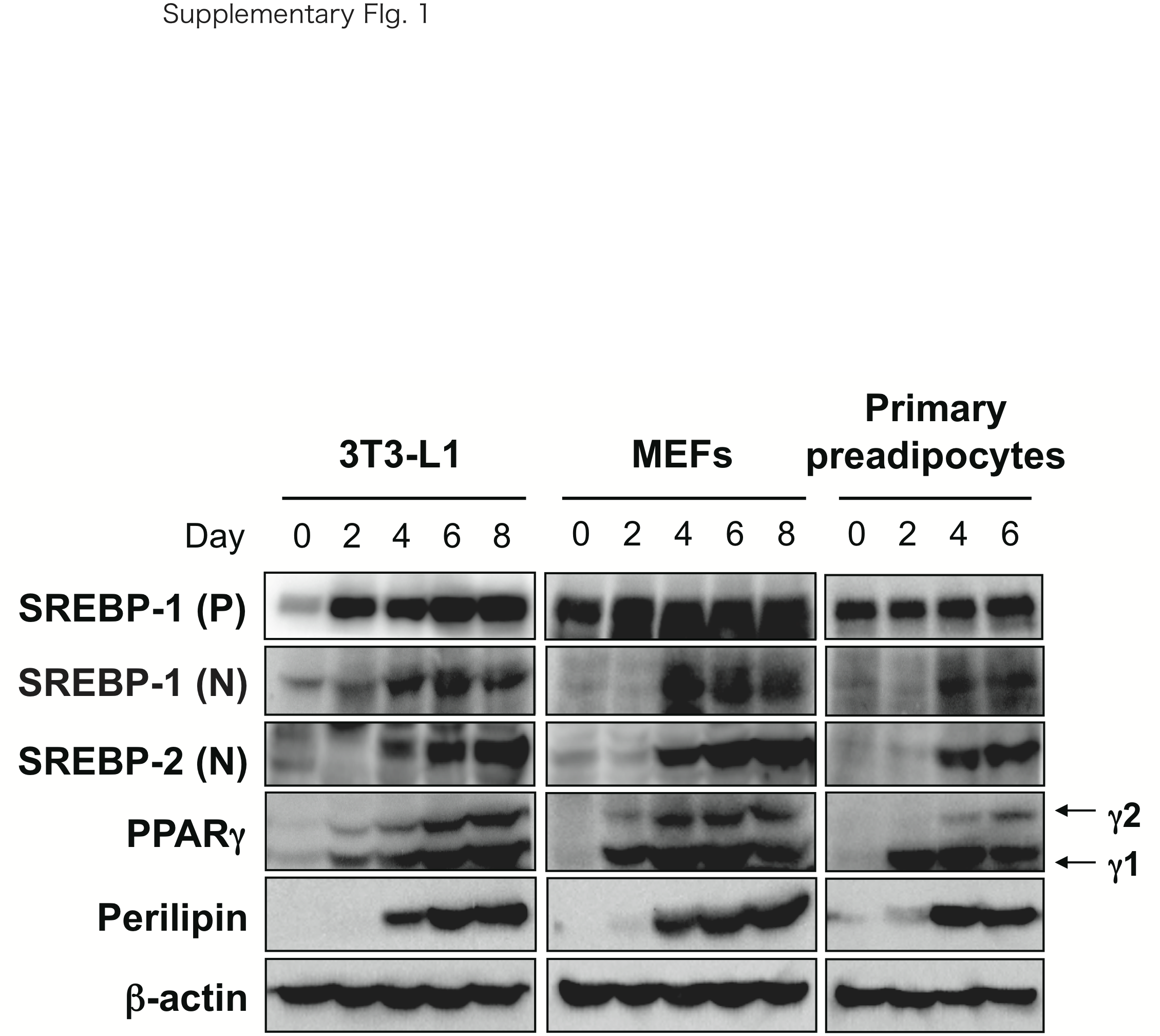

Supplement: Figure S1 — Protein expression in three types of differentiating cells. Immunoblots showing the levels of proteins in differentiating 3T3-L1 cells, MEFs and fibroblastic primary preadipocytes. P and N denote the precursor and nuclear form of SREBPs, respectively. (TIFF) [file pone.0064605.s001.tiff]

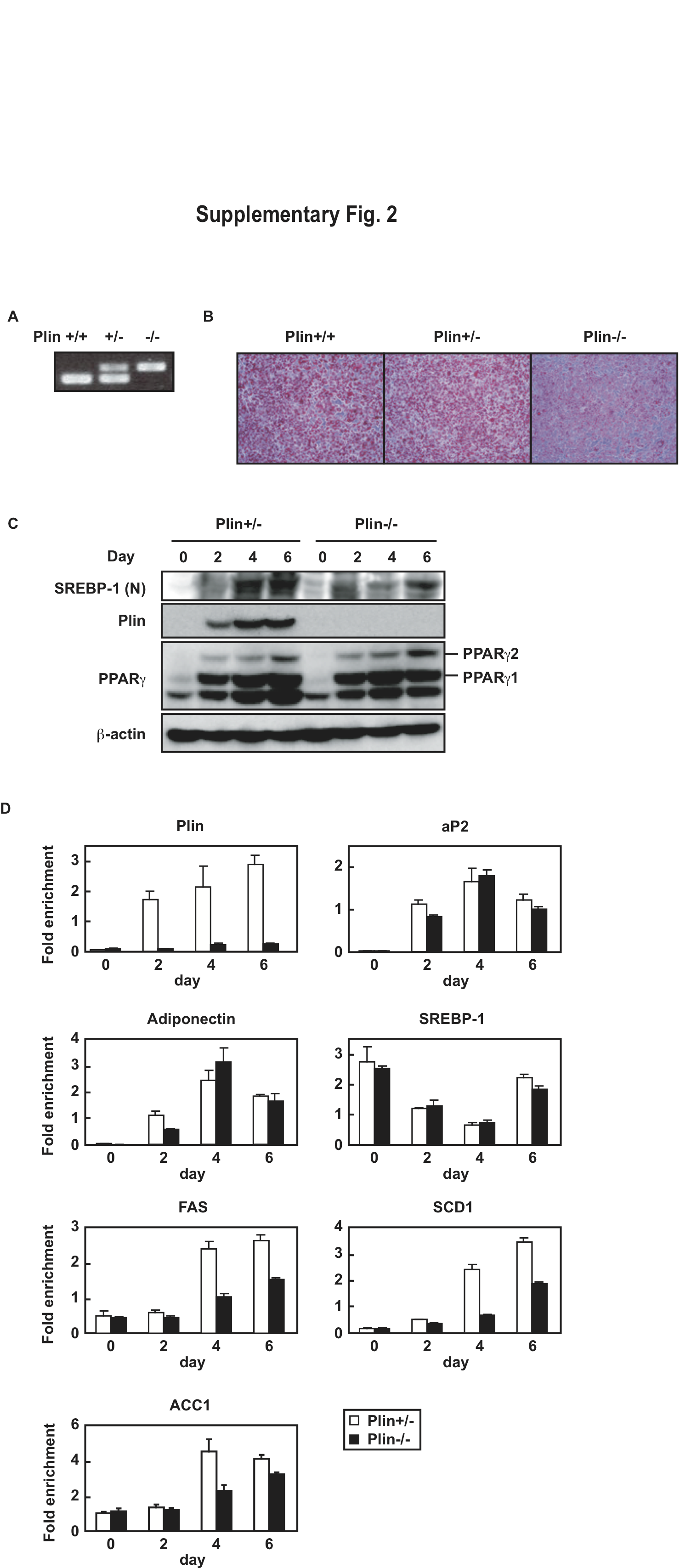

Supplement: Figure S2 — Plin deficiency suppresses TAG accumulation and SREBP-1 activation in differentiated primary adipocytes. (A) PCR analyses for genotyping. (B) Pictures of Oil Red O staining of differentiated primary adipocytes prepared from three types of mice. (C) Immunoblots showing the levels of proteins during adipogenesis of plin+/− or −/− MEFs. N denotes the nuclear form of SREBP-1. (D) Quantitative RT-PCR analyses showing the gene expression patterns in the differentiating Plin+/− or −/− MEFs. S17 rRNA was used as an internal control to normalize the mRNA level of each gene. Data are means ± SD (n = 3). (TIFF) [file pone.0064605.s002.tiff]

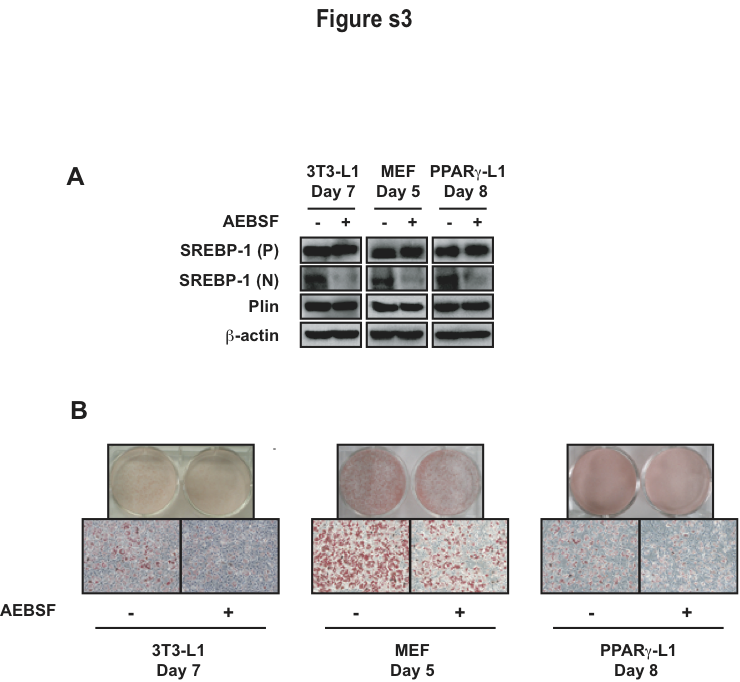

Supplement: Figure S3 — AEBSF treatment reduces intracellular TAG accumulation in differentiated cells. (A) Differentiated 3T3-L1 (day 7), MEFs (day 5) and PPARγ-expressing 3T3-L1 cells (day 8 after infection) were treated with or without 300 µM AEBSF for 24 h. Immunoblots showing the levels of proteins in three differentiated cells. P and N denote the precursor and nuclear form of SREBP-1, respectively. (B) Pictures of Oil Red O staining of these cells after the treatment. (TIFF) [file pone.0064605.s003.tiff]

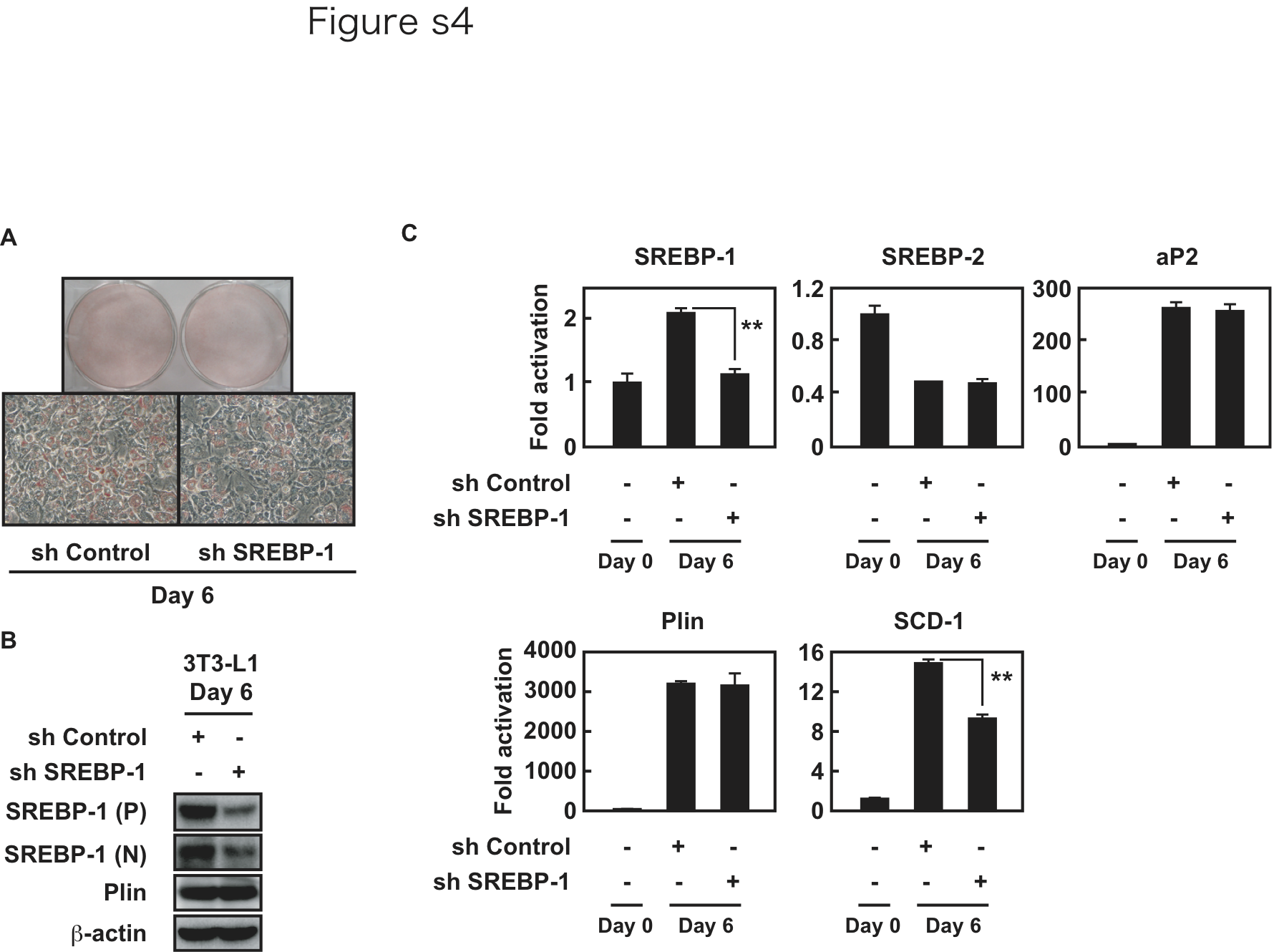

Supplement: Figure S4 — Reduced SREBP-1 expression decreases TAG accumulation along with its target gene expression in differentiated 3T3-L1 cells. 3T3-L1 cells were infected with one of lentiviral vectors expressing control shRNA or shRNA for SREBP-1 on day -1. On day 0 the cells were induced to differentiate. The lentiviral plasmid for shRNA of mouse SREBP-1 was constructed by recombining pCS-RfA-EG (RIKEN) with the pENTR4-H1 (RIKEN) inserted by oligonucleotide DNA for shRNA expression [14]. The target sequences are as follows: SREBP-1; 5′-GCGGCTGTTGTCTACCATAAG-3′, control (Scramble II Duplex from Dharmacon); 5′- GCGCGCTTTGTAGGATTCG –3′. (A) Pictures of Oil Red O staining of differentiated 3T3-L1 cells infected with one of shRNA lentivirus vectors on day 6. (B) Immunoblots showing the levels of proteins in differentiated 3T3-L1 cells. P and N denote the precursor and nuclear form of SREBP-1, respectively. (C) Quantitative RT-PCR analyses showing the gene expression patterns in differentiating 3T3-L1 cells on day 0 and 6 (n = 3). S17 rRNA was used as an internal control to normalize the mRNA level of each gene. The relative mRNA levels in the cells infected with the control virus on day 0 are considered as 1.0. **p<0.01 versus sh control. (TIFF) [file pone.0064605.s004.tiff]
